# Psychosocial Impact of COVID-19 Pandemic in Libya: A Cross-Sectional Study

**DOI:** 10.3389/fpsyg.2021.714749

**Published:** 2021-08-17

**Authors:** Alhadi M. Jahan, Marwa Mohamed, Mohammed Alfagieh, Nehal Alnawy, Muhayman Alsabiri, Rayan Algazal, Rana Saaleh, Safa El Swisy, Orjwan Abbas, Wesal Al Delawi, Butaina Abdulhafith, Osama Almangoush, Fathalla Elhag, Abdulbasit Elshukri, Wesam Abushaala, Taqwa Shahrani, Ahmed Tnton, Heba Alkilani, Amaal Dier

**Affiliations:** ^1^School of Rehabilitation Sciences, University of Ottawa, Ottawa, ON, Canada; ^2^Faculty of Dentistry, University of Benghazi, Benghazi, Libya; ^3^Faculty of Medicine, University of Misrata, Misrata, Libya; ^4^Faculty of Medicine, University of Tripoli, Tripoli, Libya; ^5^Faculty of Medicine, University of Benghazi, Benghazi, Libya; ^6^Faculty of Pharmacy, University of Misrata, Misrata, Libya; ^7^Faculty of Medical Technology, University of Tripoli, Tripoli, Libya

**Keywords:** COVID-19, pandemic, stress, self-efficacy, depression, sleep quality, Libya

## Abstract

**Background:** Since the outbreak of COVID-19 were announced in Wuhan, China, the virus has spread in most countries. After one year of restrictive measures applied by governments, it is unclear how this prolonged social distancing has affected the mental health of individuals in Libya. Therefore, the present study aims to assess the levels of perceived stress, depression, sleep disturbance, and self-efficacy associated with the pandemic in Libya and their association with the demographic factors.

**Methods:** A cross-sectional study was conducted between October 10 and November 10, 2020 in 21 cities in Libya using an online survey. The survey collected socio-demographic variables and other important psychological variables using valid scales: namely, the Perceived Stress scale, the PROMIS Depression scale, the PROMIS Sleep Disturbance scale, and self-efficacy 6-item scale. Additionally, data were collected regarding eating and smoking habits, housing and living situations, and the preparedness of the public to manage the upcoming waves of the pandemic.

**Results:** The questionnaire was accessed 746 times, and a total of 683 completed questionnaires were analyzed (response rate of 91.6%), with ages ranging from 18 to 94 years (Mean ± SD = 27.09 ± 10.57). Among the respondents, 58.4% were females and 77% were from the age group 18–29 years. The perceived stress, depression, and sleep disturbance symptoms were high in overall population. For stress, 52.7 and 17.1% of respondents reported moderate and high stress, and for sleep quality 28.8 and 8.1% reported moderate and high sleep disturbance, respectively. For depression, the overall median score was 20 (out of 40). The perceived stress, depression, and sleep disturbance were more prevalent among females and the younger age groups (18–29 years old). The overall self-efficacy median total score was 6.67 (out of 10), with a significantly higher median total score for males than females (7 vs. 6.33, *p* = 0.001). About 14.5% of the respondents were regular cigarette smokers, and most of them (79.8%) described their smoking habits during the previous month as more than typical. In addition, the reported eating habits of almost half of the respondents (43%) had changed, with about one-third (29.6%) reporting that their eating habits had become less healthy during the pandemic.

**Conclusion:** The findings of this research suggest increased levels of stress, depression, and sleep disturbances as well as COVID-19-related fear during the pandemic, especially among young females. This alarming finding urgently calls for safe and low-barrier interventions to help mentally burdened individuals. This study makes a significant contribution in providing essential data on the psychological and social impacts on the Libyan population due to the COVID-19 pandemic.

## Introduction

Since the first cases of COVID-19 were announced in Wuhan, China, in December 2019, the virus has spread rapidly in most countries worldwide (Ebada et al., 2020). In January 2021, the World Health Organization (WHO) reported almost 84 million confirmed cases worldwide, with nearly 2 million deaths (World Health Organization, 2021a). In Libya, as of today (January 4, 2021), a total of 101,414 cases have been confirmed, with 1,510 deaths (World Health Organization, 2021b). Given the fragile political situation in Libya, which has caused deteriorations in healthcare services, the rapid increase in COVID-19 has increased panic and uncertainty among the Libyan public. To date, there is no cure for COVID-19. Despite promising results regarding vaccines have been published, the pandemic will not end overnight (The Lancet Microbe, 2020). Thus, more restrictive actions are expected to be brought in for several months at best. Therefore, current recommendations in most countries involve restrictive behaviors in order to slow down the spread of the virus. Those restrictions include limitations on personal lives as well as closure of public facilities, educational institutions, and borders with neighboring countries.

These restrictive measures can contribute to adverse psychosocial outcomes such as post-traumatic stress symptoms, fear of spreading the infection to family members, anger, frustration, loneliness, denial, confusion, insomnia, and extreme behaviors, including suicide (Raza et al., 2020; Serafini et al., 2020). In a study investigating the mental health burden on the German public during the COVID-19 pandemic, Bauerle et al. (2020) reported a significant increase in the prevalence of generalized anxiety, psychological distress, and COVID-19-related fear. Similarly, in a study conducted in China by Huang and Zhao (2020), it was shown that the prevalence of generalized anxiety and poor sleep quality of the respondents were high. Interestingly, both Bauerle et al. (2020) and Huang and Zhao (2020) reported a higher prevalence of psychosocial burdens among younger people than for other segments of the population. Furthermore, Dubey et al.'s (2020) comprehensive review of the literature found psychosocial burdens similar to those reported in Germany and China, and highlighted the urgent need to set up mental health institutions to deal with current and future waves of COVID-19 outbreaks.

Recent research that investigated the psychosocial impact of COVID-19 in the UK (Shevlin et al., 2020), Italy (de Girolamo et al., 2020), Spain (Rodríguez-Rey et al., 2020), China (Huang and Zhao, 2020), India (Verma and Mishra, 2020), and the United Arab Emirates (Saddik et al., 2021) have found moderate to severe levels of stress, depression, and poor sleep quality among the public. These psychosocial burdens have been related to the mandatory restrictive measures, such as lockdowns, social distancing, lifestyle changes, public fears, and worry about the progression of the pandemic. A recent systematic review examining the psychological impacts of the quarantine during pandemics documented that at least one in five people who were exposed to restrictive measures during pandemics reported clinically significant levels of psychological burdens, mainly stress (21%) and depression (22.69%) (Cavicchioli et al., 2021). Similarly, a systematic review and meta-analysis based on seventeen studies that investigated the prevalence of psychological burdens among the general population during the COVID-19 pandemic found that the prevalence of stress in a sample size of 9,074 participants was (29.6%), and the prevalence of depression in a sample size of 44,531 participants was (33.7%) (Salari et al., 2020). The current literature on psychosocial impact of COVID-19 enriches our understanding of the different aspects of the pandemic. That is, COVID-19 pandemic does not only affect the physical health of individuals, but it also results in several psychological burdens that need immediate attention. It is worth noting, however, that the vast majority of the studies mentioned above were based on Asian or European samples; therefore, the findings of these studies may or may not apply to the Libyan scenario. In fact, there is very little research have been conducted in Arab countries in this regard; and thus, the current study sought to fill this literature gap.

Previous research also shows that the COVID-19 pandemic has had a significant impact on the public due to mandatory social distancing and stay-at-home orders, which mandate necessary lifestyle changes (Di Renzo et al., 2020). Eating and smoking habits are among the factors that have been frequently discussed in the literature as important indicators of psychosocial well-being and that have serious consequences on public health on the long run. For example, stay-at-home orders and restrictions applied to grocery stores may cause overeating and dependency on less healthy food options, which pose significant health issues. Also, the quarantine strategies can lead to stress and boredom (Droit-Volet et al., 2020), which in turn can cause greater energy intake which may be associated with dependency on “comfort food,” including high-sugar items (Moynihan et al., 2015; Rodríguez-Martín and Meule, 2015). These disturbances, beside the closures of gyms and decreased physical activity during the pandemic, can lead to severe health complications, including obesity and heart diseases (Muscogiuri et al., 2020).

Furthermore, increased smoking is another consequence of quarantine measures applied by governments. Research shows that nicotine addiction (e.g., from cigarettes) is among the risks associated with the COVID-19 pandemic (Ahluwalia et al., 2020). Individuals may use cigarette smoking as a coping strategy to relieve stress, anxiety, and depression during the pandemic (Caponnetto et al., 2020; Pfefferbaum and North, 2020); however, cigarette smoking has been linked to poor health outcomes in patients with other coronaviruses, such as Middle East Respiratory Syndrome (Park et al., 2018). A recent study in the United States found that cigarette smoking can decrease immunity, increase the risk of respiratory infections and diseases (e.g., asthma), and may cause heart and lung diseases (United States Public Health Service Office of the Surgeon General, 2020). Therefore, managing lifestyle changes related to eating and smoking during COVID-19 is imperative because both are modifiable risk factors, and once targeted they may minimize serious health problems among the public.

There is an urgent need to address the psychosocial problems caused by the COVID-19 pandemic on the public. Since this is the first nation-wide investigation of the psychosocial impacts of COVID-19 in Libya, the results of this study can provide essential data on the psychological and social impacts on the Libyan population and various factors related to mental health issues during the COVID-19 pandemic. Such data are required in order to provide much-needed evidence on current mental health issues and to provide important information regarding the need for targeted interventions to support the Libyan community.

The present study aimed to determine the psychological and social impacts of COVID-19 on the public in Libya, and to identify populations who are more vulnerable to social and mental crises associated with COVID-19 pandemic. More specific, the current study explored the levels of perceived stress, depression, sleep disturbance, and self-efficacy associated with the pandemic among adults and older adults in Libya. It also examined the association between stress, depression, sleep disturbance, self-efficacy levels, and demographics, such as age, gender, geographic location, education, and marital status. Additionally, data were collected regarding eating and smoking habits, housing and living situations, and the preparedness of the public to manage the upcoming waves of the pandemic. A more comprehensive understanding of the psychological and social burdens among different groups of the Libyan population throughout this period is urgently needed to give relevant support and thus enhance the psychosocial well-being of the community at large.

## Methods

### Study Design and Participants

To determine the psychosocial impact of COVID-19 on the general public in Libya, a cross-sectional online survey method was used (Regmi et al., 2016). The study targeted members of the general public in Libya who met the following inclusion criteria: (1) be Libyan citizens or foreigners who had lived in Libya for at least 2 years (i.e., before the beginning of the outbreak); (2) be aged 18 years or older; and (3) be able to read and understand the Arabic language. In the data analysis phase, all questionnaires that did not meet the above-mentioned criteria were excluded.

### Sample Size Calculation

The sample size was estimated by using Epi Info web-based software, version 3. Since the literature search did not retrieve similar studies conducted among the general public in Libya, we hypothesized the probability of the psychosocial burden due to COVID-19 in Libya as 50%, the confidence interval is 97%, a precision of ±5%, and a design effect of 1.0, with the minimum required sample size estimated at 471 participants. Additionally, we added 10% to the sample size to account for possible non-responses. Therefore, the minimum sample size was estimated to be 518 participants.

### Data Collection Tool

The survey by Wolf et al. (2020) was adopted and modified to meet the purpose and context of the current study. Moreover, well-known scales were incorporated into the study questionnaire: namely, the Perceived Stress Scale, the PROMIS Sleep Disturbance scale, the PROMIS Depression scale, and a 6-item self-efficacy scale, all of which are described in detail in the next section.

#### Demographic Information

Demographic variables included place of residence based on four regions of Libya (Western, Middle, Eastern, and Southern regions), gender, age, marital status, education (the highest educational degree obtained), number of family members sharing the same household, occupation, and income.

#### Perceived Stress Scale (PSS)

The Perceived Stress 10-items Scale (PSS10) developed by Cohen et al. (1983) is a widely used self-administered measure to assess the degree to which situations in one's life are described as “stressful.” It is a measure of to what degree individuals have considered their lives as being unpredictable, uncontrollable, and overloaded over the previous month. The updated version of the scale includes 10 items, and the psychometric properties of this scale have been evaluated empirically in previous research with promising results (Cronbach's α ranges from 0.70 to 0.91), which supports its use in measuring perceived stress among the general public (Roberti et al., 2006; Lee, 2012). Based on the PSS, we asked a set of 10 questions about feelings and thoughts during the previous month. In each question, participants were asked how often they felt a certain way. Each question involved five choices scored from 0 to 4 (0 = Never, 1 = Almost Never, 2 = Sometimes, 3 = Fairly Often, 4 = Very Often), with the total score being computed by adding up the scores for each item. The total possible scores ranged from 0 to 40, with higher scores indicating higher perceived stress. The total scores can be interpreted according to three categories: low stress (scores ranging from 0 to 13), moderate stress (scores ranging from 14 to 26), and high stress (scores ranging from 27 to 40) (Cohen et al., 1983). The original scale was developed in the English language, and therefore, we used the Arabic version of the PSS, which had been translated and validated by Chaaya et al. (2010) with a Cronbach's α of 0.74.

#### PROMIS Sleep Disturbance Scale

To evaluate the degrees of sleep disturbance and quality among respondents, we used the short form of the Patient-Reported Outcomes Measurement Information System (PROMIS) Sleep Disturbance scale version 8b (PROMIS, 2021). The PROMIS Sleep Disturbance scale consists of 8 items: sleep disturbance, sleep satisfaction, refreshing sleep, difficulty falling asleep, trouble staying asleep, trouble sleeping, getting enough sleep, and sleep quality. Respondents rated their sleep during the previous 7 days on 5-point scales. The first four items used a scale that included “*not at all,” “a little bit,” “somewhat,” “quite a bit,”* and “*very much'*; items 5, 6, and 7 used a scale comprising “*never,” “rarely,” “sometimes,” “often,”* and “*always'*; and the last item (item number 8), which assessed the overall sleep quality, used a scale of “*very poor,” “poor,” “fair,” “good,”* and “*very good*.” After obtaining permission from its developers, we used the Arabic version of this scale, which had been translated by the developers and further validated by Mahmoud et al. (2019) with a Cronbach's α of 0.70. The psychometric properties of the PROMIS Sleep Disturbance Scale are well-established in the literature (Buysse et al., 2010; Full et al., 2019).

#### PROMIS Depression Scale

The PROMIS Depression scale item bank contains 28 self-rated Likert scale items focused on four elements: negative mood, negative self-image, worthlessness, negative social cognition, and decreased positive interest in life. For the current research, the 8-item (version 8a) short form of the scale was used. The items depend on a 7-day recall period, and responses were rated on a 5-item Likert scale: (1) never, (2) rarely, (3) sometimes, (4) often, and (5) always. The scores were then converted to T-scores by using a conversion table in the PROMIS scoring manual, with higher scores indicating greater severity of depression (PROMIS, 2021). The original scale was developed in the English language (Cella et al., 2007); therefore, we used the validated Arabic version (Cronbach's α = 0.74) with permission from its developers (PROMIS, 2021).

#### Self-Efficacy 6-Item Scale

Self-efficacy refers to the belief that a person can make behavioral changes necessary to reach a pre-determined goal, even during unpredictable and stressful situations (Bandura, 1989; Clark and Dodge, 1999). The concept of self-efficacy was first theorized by Bandura in the Social Cognitive Theory, in which self-efficacy is considered a significant determinant of behavioral change and a mediating factor for self-management skills during diseases (Bandura, 1998). To control the spread of COVID-19, individuals should make behavioral changes that can influence their everyday life activities. In this study, we used the Self-Efficacy 6-Item Scale (SE6S) to measure how confident people were in adopting preventative behaviors during certain activities. The measure is available for use without permission, and it consists of 6 items that are rated on a 10-point scale that ranges from (1) “not at all confident” to (10) “totally confident.” The total score of the scale is calculated by computing the mean scores of at least four of the six items, with higher numbers indicating greater self-efficacy (Lorig et al., 1989). We used this scale based on the assumption that self-management skills are central in avoiding infection and preventing the spread of COVID-19 in the community. There is growing evidence that supports the use of the SE6S as a valid and reliable measure of perceived self-efficacy in many conditions that require self-management skills, such as diabetes (Allam et al., 2020) and hypertension (Hu et al., 2013). The original version of the SE6S was developed in English by Lorig et al. (1989); therefore, for the current study, we used the Arabic version of the instrument that was translated and validated (Cronbach's α = 0.79) by Allam et al. (2020).

#### Eating and Smoking Habits

We assessed changes in the eating habits of the respondents by asking this simple question: “Do you think your eating habits have changed after the pandemic?,” with responses being either “yes” or “no.” Then, a follow-up question was asked: “Do you think your eating habits have become more healthy, less healthy, or stayed the same?” The responses ranged from: (1) more healthy, (2) less healthy, (3) stayed the same, or (4) cannot tell. To assess changes in smoking habits among the participants, we first asked this question: “In the last month were you smoking cigarettes regularly?,” with responses being “yes” or “no.” Then, we asked about the average number of cigarettes the respondents smoked per day. The responses were anchored on three responses: <1 pack, 1 pack or more, or cannot tell (where 1 pack = 20 cigarettes). To assess whether there had been a change in smoking habits due to the pandemic, we asked this question: “Thinking about the last month, would you say this was more, less, or the same than what is typical for you?,” with corresponding responses.

#### Housing and Living Situation

Given the intermittent military conflicts in Libya, which have resulted in thousands of families fleeing their homes to a safer place (Human Rights Watch, 2020), and the acknowledged association between housing and living situation regarding the spread of infection and poor health outcomes (Ahmad et al., 2020), we asked questions to assess the housing and living situations of respondents. Respondents answered questions first about the number of family members who shared the same household, and then they answered two questions about their living situation with regard to whether they still lived in their homes or had fled due to war or pandemic reasons.

#### Preparedness

To examine the perceived preparedness of respondents, we asked about their levels of preparedness in case another outbreak were to occur, with the answers being: very prepared, somewhat prepared, a little prepared, or not prepared at all. Respondents were also asked about their confidence in their local municipalities and the government in preventing further outbreaks of COVID-19. The answers here were: very confident, somewhat confident, not very confident, or not confident at all. Also, respondents answered “yes” or “no” to questions about the challenges they faced either at home or at work in maintaining social distancing measures.

### Pilot Testing

After three rounds of discussion with the research team, the preliminary version of the questionnaire was approved and tested on 23 volunteers in order to assess the questions' clarity and the time required to complete the questionnaire. Data obtained during the pilot-testing phase were not included in the data analysis for this study. Based on the pilot-testing, the questions were determined to be clear and easy to understand by different age groups.

### Data Collection

The final version of the survey was circulated through several online channels, including social media platforms (e.g., Facebook and Twitter), the official social media pages of sports clubs, universities, healthcare institutions, and community associations, and the official sites of 21 municipalities across the country representing four geographical regions of Libya: the Western, the Middle, the Eastern, and the Southern regions. Further, to speed up the data collection process, a snowball sampling procedure was used. The study's authors and collaborators invited their family members, friends, and colleagues to publish links to the study in their professional networks. The survey was made accessible for a period of 1 month (from October 10 to November 10, 2020).

### Ethical Statement

This study was approved by the Research Ethics Committees of the University of Tripoli, the University of Misrata, the National Cancer Institute (Misrata), and Misrata Medical Centre [Ethics certificate# M.A. 2-4/354]. Also, electronic informed consent was obtained from all participants before proceeding to the study questionnaire. Participation was entirely voluntary and anonymous, and the questions were formatted in a way that allowed for withdrawal at any time. To the best of our knowledge, all study procedures were conducted in accordance with the Declaration of Helsinki (World Medical Association, 2013).

### Data Analysis Plan

After checking for data normality, descriptive statistics were calculated for all participants' characteristics and survey responses. Dichotomous variables were expressed as frequency and percentage, while continuous variables were expressed as mean ± standard deviation or median ± interquartile range (IQR) in the cases of normally and not-normally distributed data, respectively. For comparison of the categorical variables, we used the Chi-square test. Associations between participant characteristics and responses to the COVID-19 impacts on psychosocial variables were examined in bivariate analyses using the Mann-Whitney *U* test or Kruskal-Wallis H test, as appropriate. Spearman rank correlations were used in the correlation analysis. Also, multivariate linear regression analysis was performed to explore potential influence factors for stress, sleep quality, depression, and self-efficacy during the pandemic. All data were analyzed using the Statistical Package for Social Sciences (SPSS) version 26.0. An alpha level below 0.05 was considered for statistical significance (2-sided tests).

## Results

### Demographic Characteristics

The online survey was accessed 746 times, with a total of 683 completed questionnaires being included in the final analysis (response rate of 91.6%). Of these 683 respondents, there were 399 females (58.4% of the total sample), resulting in an overall male to female respondent ratio of 0.7:1. The responses came from four geographic regions of Libya: the Western, Middle, Eastern, and Southern regions. A total of 240 (35.1%) of respondents were from the Western region, 307 (44.9%) were from the Middle region, 84 (12.3%) were from the Eastern region, and 52 (7.6%) were from the Southern region. Among the respondents, 526 (77%) were from the age group 18–29 years old, followed by 74 from the age group 30–39 (10.8%), 45 from the age group 40–49 years old (6.6%), and 25 from the age group 50–59 years old (3.7%), with only 13 of the respondents being 60 years of age or over (1.9%). The age range of the respondents was 18–94 years, and the mean age in years ± Standard Deviation was Mean ± SD = 27.09 ± 10.57.

Most participants (557; i.e., 81.6%) were single, while 119 (17.4%) were married. Regarding level of education, the majority 346 (50.7%) held secondary school certificates, 245 (35.9%) had bachelor's degrees, and 51 (7.4%) held graduate and/or postgraduate qualifications. The most frequent response (49.2%) for family members living in the same household was “5–7,” with 227 (33.2%) stating more than 7 family members, and 110 (16.1%) stating households of 2–4 members. A total of 425 (62.2%) of the respondents were students, 103 (15.1 %) were public sector employees, 95 (13.9%) were private sector employees, 45 (6.6%) were either not working, unemployed, or were homemakers, and 15 (2.2%) were retired. In terms of monthly income as measured by the Libyan dinar (LD) (1 Libyan Dinar equals ~0.22 United States Dollars) (Central Bank of Libya, 2021), the highest proportion of respondents (33.4%) stated monthly incomes of 1,000–2,000 LD, while 4% stated “ <500 LD” as monthly income (see Table 1 for details).

**Table 1:** Demographic characteristics of study sample (*N* = 683).

**Demographics**	***N***		**Frequency**	**%**
Gender	683	Male	284	41.6
		Female	399	58.4
Age	683	18–29	526	77.0
		30–39	74	10.8
		40–49	45	6.6
		50–59	25	3.7
		60+	13	1.9
		**Range (18–94 years)** **Mean (27.09 ± 10.57)**		
Geographic location	683	Western region	240	35.1
		Middle region	307	44.9
		Eastern region	84	12.3
		Southern region	52	7.6
Marital status	682	Single	556	81.4
		Married	119	17.4
		Divorced	5	0.7
		Widowed	2	0.3
Education	683	Primary	28	4.1
		Secondary	346	50.7
		Bachelor	245	35.9
		Master or higher	51	7.4
		Vocational training	7	1.0
		No formal education	6	0.9
Occupation	683	Student	425	62.2
		Public sector employee	103	15.1
		Private sector employee	95	13.9
		Not working, unemployed, housewife	45	6.6
		Retired	15	2.2
Monthly income	683	<500 LD	27	4.0
		500–1,000 LD	226	33.1
		1,000–2,000 LD	228	33.4
		2,000–5,000 LD	143	20.9
		More than 5,000 LD	59	8.6

### Prevalence of Perceived Stress, Sleep Disturbance, and Depression During COVID-19 Pandemic in the Overall Population

In this work, the prevalence of perceived stress among general population during the COVID-19 pandemic was high across the three categories, i.e., low stress (total scores ranging from 0 to 13), moderate stress (total scores ranging from 14 to 26), and high stress (total scores ranging from 27 to 40). More than half of the respondents (52.7%, *N* = 360) reported moderate stress, while 17.1% (*N* = 117) reported having high stress. However, almost one-third of the population reported having low stress (30.2%, *N* = 206). Similarly, the assessment of sleep during the COVID-19 outbreak revealed overall poor sleep quality among the respondents, with more than half (51%) of the respondents reporting mild, moderate, or severe sleep disturbances (i.e., 13.2, 28.8, and 8.1%, respectively). The following subsections presents all the above scores stratified by gender and age.

### Prevalence of Perceived Stress, Sleep Disturbance, Depression, and Self-Efficacy Stratified by Gender

**Figure 1:**
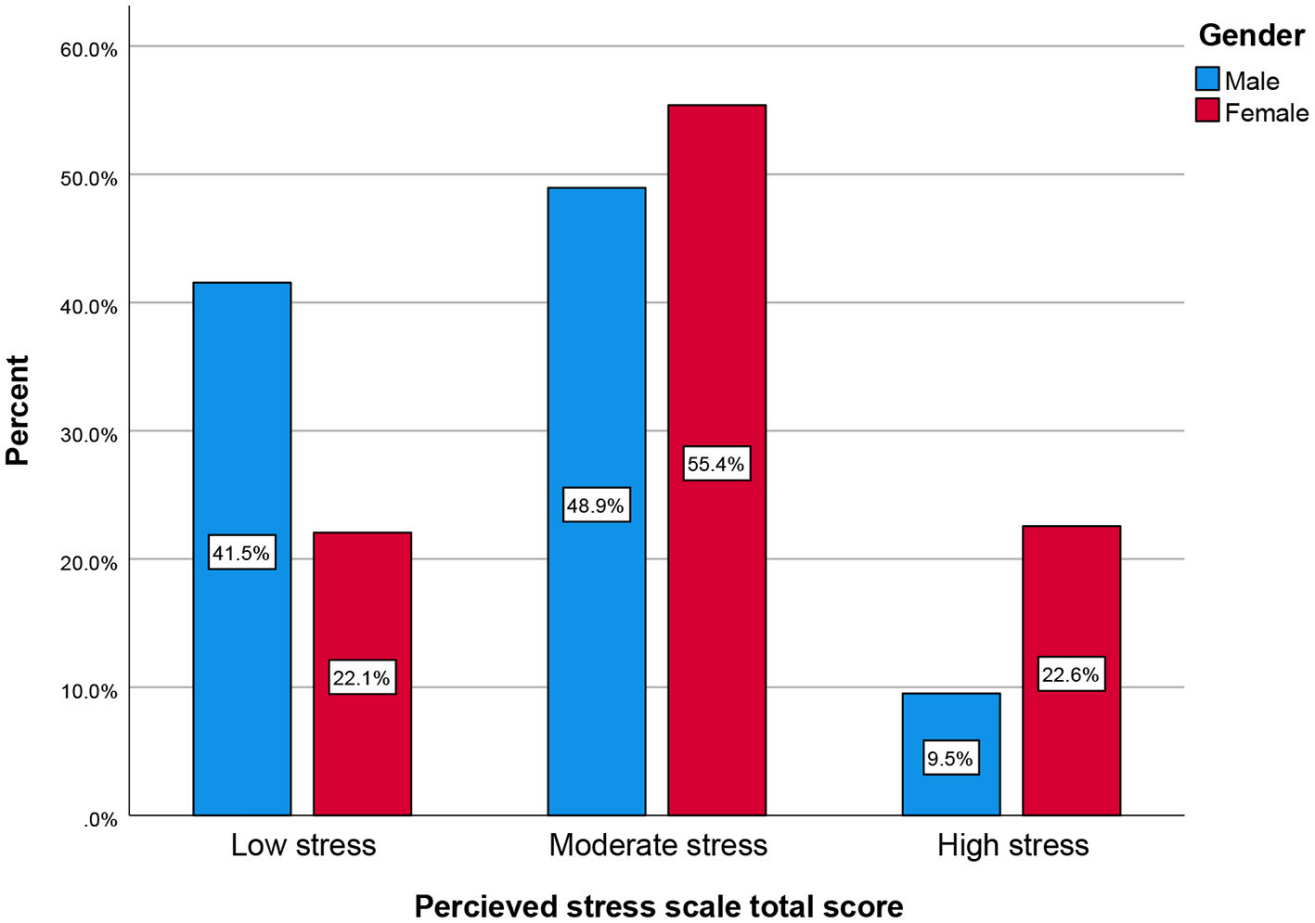
Perceived stress total score by gender (cluster bar count).

**Figure 2:**
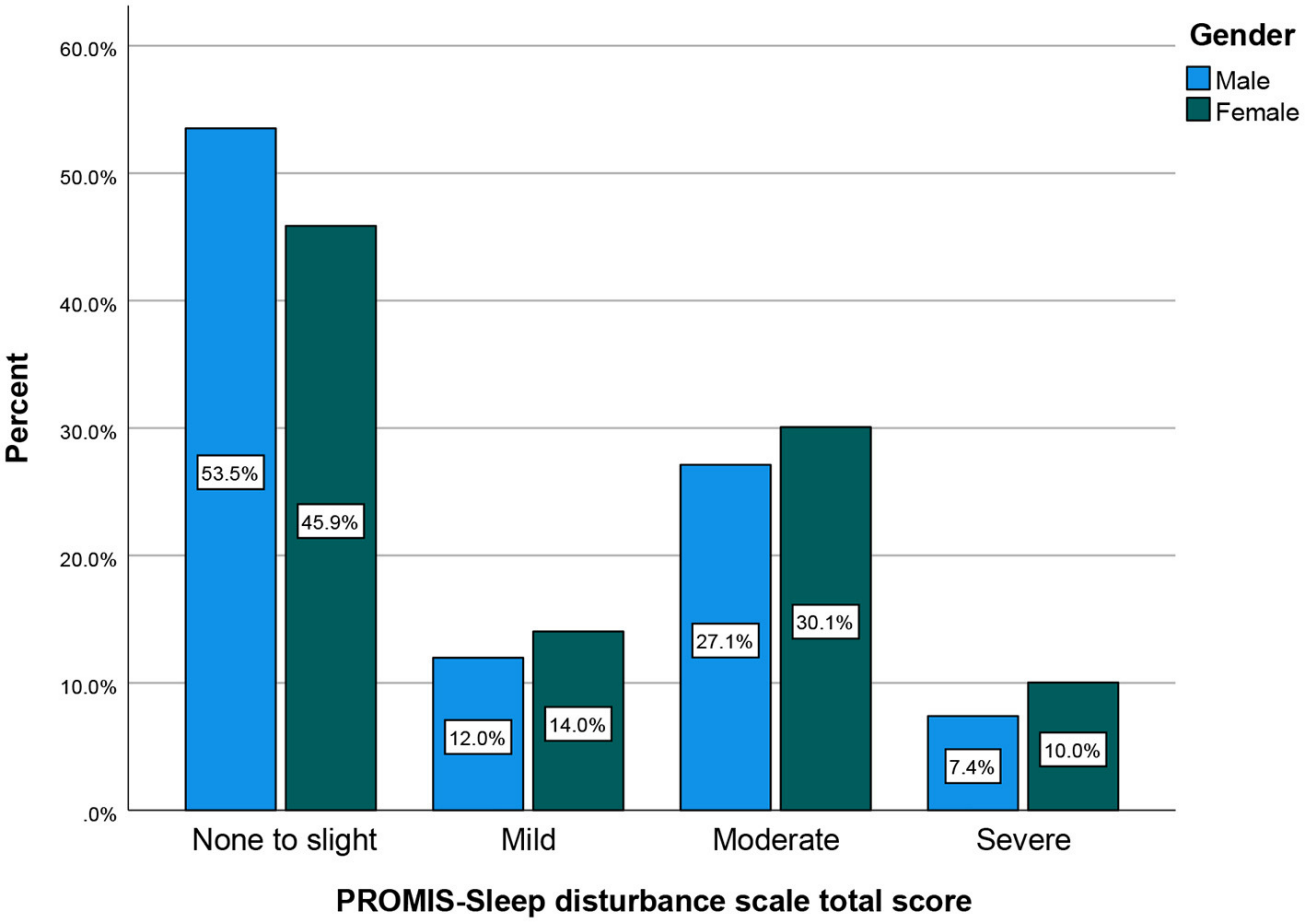
Sleep disturbance total score by gender (cluster bar count).

**Figure 3:**
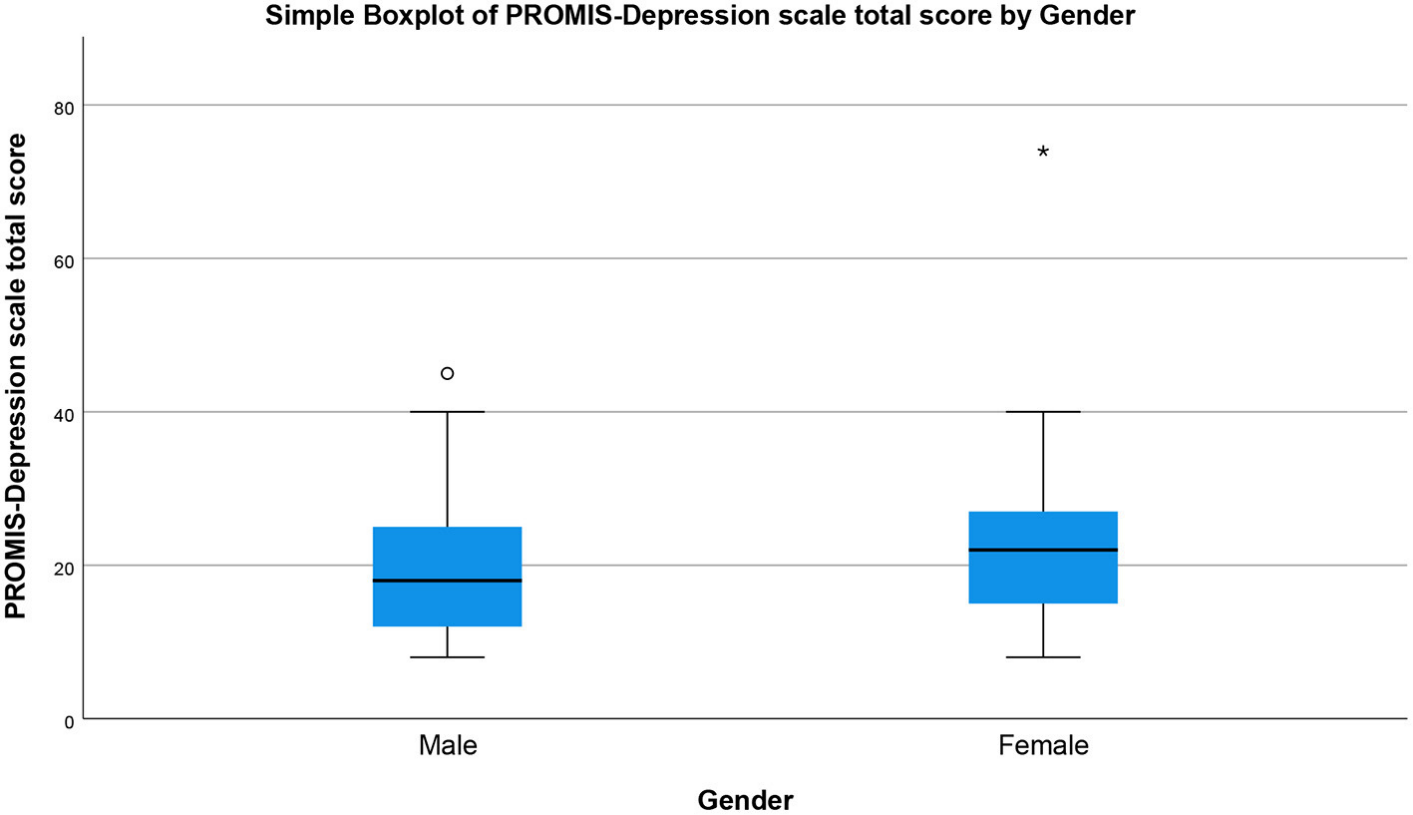
Depression total score by gender (simple boxplot).

**Figure 4:**
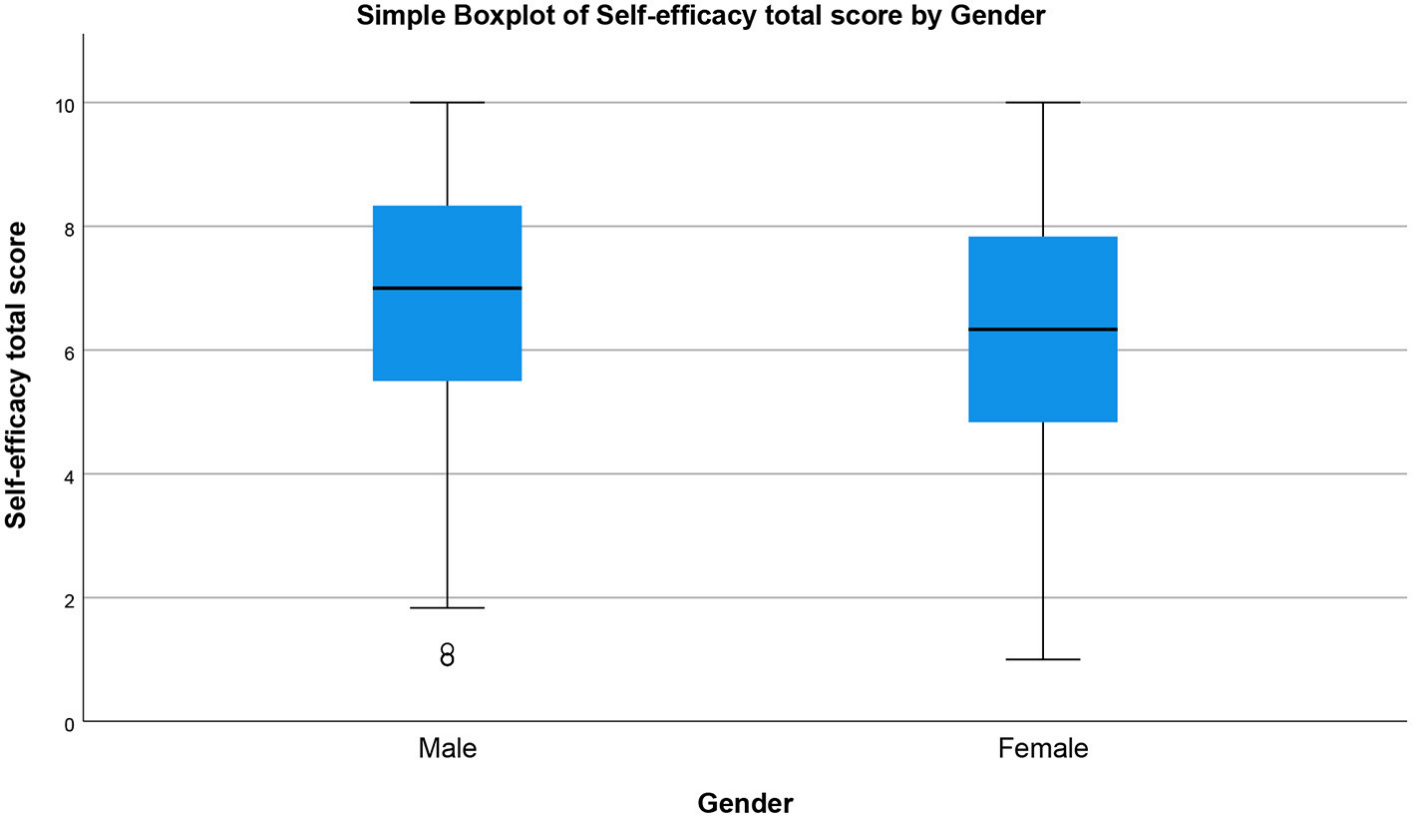
Self-efficacy total score by gender (simple boxplot of mean).

The prevalence of perceived stress, sleep disturbance, depression, and self-efficacy during COVID-19 pandemic by gender are presented in Figures 1–4, respectively (see Supplementary File 1). There was a statistically significant difference in the prevalence of all of the above-mentioned variables by gender (*P* < 0.05, as shown in Table 2).

**Table 2:** Prevalence of perceived stress, sleep disturbance, depression, and self-efficacy stratified by gender (*N* = 683).

**Variables**	**Total** **(***n*** = 683)**	**Male** **(***n*** = 284)**	**Female** **(***n*** = 399)**	**Mann-Whitney** **U**	***Z***	**Signature**
Perceived stress scale total score, Median (IQR)	18.00 (12)	15 (10)	20 (12)	39775.5	−6.648	0.001^^¶^^
PROMIS-Sleep disturbance scale total score, Median (IQR)	56.30 (17)	54.3 (17.1)	56.3 (15.8)	51078.5	−2.197	0.028^^¶^^
PROMIS-Depression scale total score, Median (IQR)	20.00 (12)	18 (13)	22 (12)	45,726	−4.305	0.001^^¶^^
Mean Self-efficacy total score, Median (IQR)	6.6667 (3)	7 (2.83)	6.33 (3)	46,635	−3.945	0.001^^¶^^

¶ *Significant p-value*.

Notably, the prevalence of perceived stress as measured by the Perceived Stress Scale (PSS) was relatively high for both males and females, although females showed significantly higher stress in both the moderate stress and high stress categories at 32.36% (*N* = 221) and 13.18% (*N* = 90), respectively. For the low stress category, however, males showed more stress at 17.28% (*N* = 118) compared to females 12.88% (*N* = 88) (see Figure 1).

Further, sleep quality as measured by the PROMIS Sleep Disturbance scale showed significant differences between males and females, as shown in Figure 2. Sleep disturbance was stratified by severity of: none to slight, mild, moderate, or severe. Female respondents reflected higher sleep disturbance scores in all four categories, at 26.79% (*N* = 183), 8.2% (*N* = 56), 17.57% (*N* = 120), and 5.86% (*N* = 40) for the categories none to slight, mild, moderate, and severe sleep disturbance, respectively.

Next, we examined gender differences in self-rated depression using the 8-item PROMIS Depression scale. As presented in Table 2, there was a statistically significant difference (*p* = 0.001) between males and females in total depression scores, with median = 18, IQR = 13, *N* = 284 for male respondents and median = 22, IQR = 12, *N* = 399 for female respondents. Figure 3 presents the gender differences of the total depression score by employing a simple boxplot.

In terms of self-efficacy scores, as measured by Self-Efficacy 6-Item scale, there was a significant difference between male and female respondents (*p* = 0.001) (see Table 2). As shown in Figure 2, the mean total score for self-efficacy was higher in males (median = 7, IQR = 2.83, *N* = 284) compared to females (median = 6.33, IQR = 3, *N* = 399).

### Prevalence of Perceived Stress, Sleep Disturbance, Depression, and Self-Efficacy Stratified by Age Groups

The prevalence of perceived stress, sleep disturbance, depression, and self-efficacy stratified by age groups are shown in Table 3. Here, the youngest age group (18–29 years) reported the highest total scores for perceived stress, sleep disturbance, and depression. Interestingly, the same age group (i.e., 18–29 years) reported the lowest total scores for self-efficacy.

**Table 3:** Perceived stress, sleep disturbance, depression, and self-efficacy stratified by age (*N* = 683).

**Variables**	**18–29 years** **(***n*** = 526)**	**30–39 years** **(***n*** = 74)**	**40–49 years** **(***n*** = 45)**	**50–59 years** **(***n*** = 25)**	**60+ years** **(***n*** = 13)**	**Kruskal-Wallis** **H (df)**	**Sig**.
Perceived stress scale total score, Median (IQR)	19.00 (12.00)	17.00 (10.25)	17.00 (12.50)	14.00 (11.00)	13.00 (17.50)	15.914 (4)	0.003^¶^
PROMIS-sleep disturbance scale total score, Median (IQR)	57.30 (14.70)	54.30 (15.43)	47.90 (10.45)	52.20 (14.35)	44.20 (28.95)	27.478 (4)	0.001 ^¶^
PROMIS-depression scale total score, Median (IQR)	21.00 (12.00)	17.00 (12.00)	16.00 (12.00)	15.00 (11.50)	16.00 (15.00)	21.127 (4)	0.001 ^¶^
Mean self-efficacy total score, Median (IQR)	6.50 (2.83)	6.92 (3.33)	7.00 (3.33)	7.00 (3.33)	6.67 (4.75)	1.971 (4)	0.741

¶ *significant p-value*.

### Association of Influence Factors With Stress, Sleep Quality, Depression, and Self-Efficacy During COVID-19 Outbreak

As presented in Table 4, there is a significant correlation between stress, sleep quality, depression and variables of age, gender, marital status, education, and occupation. Self-efficacy, however, is associated only with gender and monthly income. We did not find any influence of family size (i.e., number of family members) on the above variables.

**Table 4:** Association of sociodemographic variables with stress, sleep quality, depression, and self-efficacy based on the bivariate regression analysis.

**Socio-demographic** **variables**		**Perceived stress scale total score**		**PROMIS-sleep disturbance scale total score**		**PROMIS-depression scale total score**		**Mean self-efficacy total score**	
Age	Spearman's rho	**−0.148**	^***^	**−0.187**	^***^	**−0.173**	^***^	0.041	
	*p*	<0.001		<0.001		<0.001		0.286	
	Kendall's tau B	**−0.119**	^***^	**−0.151**	^***^	**−0.139**	^***^	0.032	
	*p*	<0.001	<0.001	<0.001	<0.001	<0.001	<0.001	0.287	
Gender	Spearman's rho	**0.255**	^***^	**0.084**	^*^	**0.165**	^***^	**−0.151**	^***^
	*p*	<0.001		0.028		<0.001		<0.001	
	Kendall's tau B	**0.212**	^***^	**0.07**	^*^	**0.137**	^***^	**−0.125**	^***^
	*p*	<0.001		0.028		<0.001		<0.001	
Marital status	Spearman's rho	**−0.148**	^***^	**−0.179**	^***^	**−0.219**	^***^	−0.006	
	*p*	<0.001		<0.001		<0.001		0.87	
	Kendall's tau B	**−0.122**	^***^	**−0.148**	^***^	**−0.181**	^***^	−0.005	
	*p*	<0.001		<0.001		<0.001		0.875	
Education	Spearman's rho	**−0.124**	^**^	**−0.127**	^***^	**−0.153**	^***^	0.072	
	*p*	0.001		<0.001		<0.001		0.061	
	Kendall's tau B	**−0.098**	^**^	**−0.1**	^***^	**−0.121**	^***^	0.057	
	*p*	0.001		<0.001		<0.001		0.058	
Family members	Spearman's rho	−0.01		0.048		0.067		−0.03	
	*p*	0.791		0.211		0.079		0.433	
	Kendall's tau B	−0.008		0.038		0.053		−0.024	
	*p*	0.791		0.212		0.079		0.424	
Occupation	Spearman's rho	**−0.133**	^***^	**−0.112**	^**^	**−0.112**	^**^	−0.002	
	*p*	<0.001		0.003		0.003		0.969	
	Kendall's tau B	**−0.104**	^***^	**−0.088**	^**^	**−0.089**	^**^	−0.001	
	*p*	<0.001		0.003		0.003		0.979	
Monthly income	Spearman's rho	−0.065		−0.074		−0.043		**0.1**	^**^
	*p*	0.087		0.054		0.265		0.009	
	Kendall's tau B	−0.05		−0.057		−0.032		**0.077**	^**^
	*p*	0.086		0.052		0.273		0.008	

* * means p-value < 0.05*,

** * means p-value < 0.01*,

*** *means p-value < 0.001. The bold values refer to statistically significant values*.

In the multivariate regression analysis, the correlations mentioned above weakened; however, there was still statistical significance. Female participants were more likely to have higher stress than their male counterparts (*p* < 0.001). In terms of sleep quality, participants in the age group between 40 and 49 were more likely to have poorer sleep quality than the other age groups (*p* = 0.04). For the total depression scores, our analyses revealed a statistically significant association between gender and depression. Female participants were more likely to have higher depression compared to male participants (*p* < 0.001). Besides, married participants were more likely to have a higher risk for depression than those who are single, divorced, or widowed (*p* < 0.001). Similarly, we found a statistically significant association between self-efficacy total scores and gender. Female participants were more likely to have lower self-efficacy than males (*p* < 0.001). See Table 5 for details.

**Table 5:** Association of socio-demographic variables with stress, sleep quality, depression, and self-efficacy based on the multivariate regression analysis.

**Predictor**	**Estimate**	**SE**	***t***	***p***
**Model coefficients–The perceived stress**				
Intercept	16.83	1.63	10.32	<0.001
**Gender**				
Male		**References**		
Female	3.46	0.64	5.42	<0.001
**Model coefficients–sleep quality**				
Intercept	57.71	2.30	25.05	<0.001
**Age**				
18–29		**References**		
30–39	−0.03	1.77	−0.02	0.99
40–49	**−4.58**	**2.18**	**−2.10**	**0.04**
50–59	−2.69	2.72	−0.99	0.32
60+	−5.96	6.03	−0.99	0.32
**Model coefficients–depression**				
Intercept	21.83	1.76	12.42	<0.001
**Gender**				
Male		**References**		
Female	**2.04**	**0.69**	**2.97**	**<0.001**
**Marital status**				
Single		**References**		
Married	**−4.47**	**1.15**	**−3.89**	**<0.001**
Divorced	1.87	3.85	0.49	0.63
Widow	1.71	6.02	0.28	0.78
**Model coefficients–self-efficacy**				
Intercept	6.86	0.40	17.32	<0.001
**Gender**				
Male		**References**		
Female	**−0.53**	**0.16**	**−3.45**	**<0.001**

*The bold values refer to statistically significant values*.

### Smoking and Eating Changes During COVID-19 Outbreak

As presented in Table 6, a total of 683 participants responded to this section of the questionnaire. Most respondents were either non-regular cigarette smokers or non-smokers (85.5%, *N* = 584), while only (14.5%, *N* = 99) were regular cigarette smokers. Among the regular smokers, about two-thirds (63.6%, *N* = 63) smoked more than one pack of cigarettes (i.e., more than 20 cigarettes) per day. Moreover, the majority of smokers (79.8%, *N* = 79) described the number of cigarettes they had smoked in the previous month as greater than typical. In terms of eating habits, 43% (*N* = 294) of the respondents reported that their eating habits had changed after the pandemic. While a quarter of the respondents reported their eating habits had become more healthy, about 29.6% reported that their eating habits had become less healthy, as shown in Table 6.

**Table 6:** Smoking and eating habits (*N* = 683).

**Survey questions**	***N***	**Responses**	**Frequency**	**%**
In the last month were you smoking cigarettes regularly?	683	Yes	99	14.5
		No	584	85.5
On average how many cigarettes do you or did you smoke a day? (1 pack = 20 cigarettes)	99	<1 pack	27	27.3
		1 pack or more	63	63.6
		Cannot tell	9	9.1
Thinking about the last month, would you say this was more, less, or the same than what is typical for you?	99	More than typical	79	79.8
		Less than typical	9	9.1
		The same	11	11.1
Do you think your eating habits have changed after the pandemic?	683	Yes	294	43.0
		No	388	56.8
Do you think your eating habits have become more healthy, less healthy, or stayed the same??	294	More healthy	74	25.2
		Less healthy	87	29.6
		Stayed the same	21	7.1
		Can't tell	112	38.1

### Housing and Living Situations of Respondents

Using the data on the housing and living situations, we found that more than half of the study respondents (56.4%, *N* = 385) had between 6 and 10 people living in the same home, while more than a quarter of the study sample shared the same household with more than 10 people (28.9%, *N* = 198). We also found that nearly half of the respondents (42.6%, *N* = 291) had moved or changed their residences because of the war. However, only 2.1%, (*N* = 14) had changed their residence because of the pandemic (see Table 7).

**Table 7:** Housing and living situations (*N* = 683).

**Survey questions**	***N***	**Response**	**Frequency**	**%**
Today how many people other than yourself live in your home?	683	5 people or less	90	13.2
		6–10 people	385	56.4
		More than 10	198	28.9
		Do not know	10	1.5
Have you moved or changed your residence because of the war?	683	Yes	291	42.6
		No	392	57.4
Since the beginning of the pandemic, have you moved or changed your residence?	683	Yes	14	2.1
		No	669	98.0

### Preparedness for COVID-19 Outbreak

As shown in Table 8, respondents were generally unprepared for any upcoming outbreaks, as about three-quarters of them reported being “not prepared at all” or “a little prepared” for another widespread COVID-19 outbreak. Similarly, most respondents said that they were “not confident at all” or “not very confident” that the local and the central governments could prevent further outbreaks of COVID-19.

**Table 8:** Preparedness for COVID-19 outbreak (*N* = 683).

**Survey questions**	***N***	**Responses**	**Frequency**	**%**
How prepared do you think you are for another widespread coronavirus outbreak?	683	Not prepared at all	105	15.4
		A little prepared	411	60.2
		Somewhat prepared	1	0.1
		Very prepared	166	24.3
How confident are you that your local municipality can prevent further outbreak of the coronavirus?	683	Not confident at all	318	46.6
		Not very confident	278	40.7
		Somewhat confident	80	11.7
		Very confident	7	1.0
How confident are you that the Libyan government can prevent further outbreak of the coronavirus?	683	Not confident at all	385	56.4
		Not very confident	225	32.9
		Somewhat confident	69	10.1
		Very confident	4	0.6
How confident are you in being able to maintain social distance?	683	Not confident at all	141	20.6
		Not very confident	248	36.3
		Somewhat confident	219	32.1
		Very confident	75	11.0
Does your work make it difficult for you to maintain social distance?	683	Yes	419	61.3
		No	264	38.7
Does your living situation make it difficult for you to maintain social distance?	683	Yes	305	44.7
		No	378	55.3

## Discussion

We conducted this cross-sectional study to assess the perceived stress, depression, and sleep disturbances during the lockdown due to COVID-19 among the general public in Libya through an online nationwide survey. We also sought to assess the levels of subjective self-efficacy, because this has been reported as an essential indicator of self-management strategies during pandemics, and it has also been connected to psychological well-being (Xiong et al., 2020). Since eating and smoking habits have been associated with psychological well-being (Caponnetto et al., 2020; Giacalone et al., 2020), our survey included questions to assess any changes during the pandemic. Other psychosocial variables were also considered in this survey, such as housing situations, preparedness, and trust in local and central governments to manage the ongoing and upcoming waves of the COVID-19 virus.

### Summary of Main Findings

A total of 683 respondents were included in our study. Of them, 399 participants (58.4%) were females, 526 participants (77.0%) were aged between 18 and 29 years, 556 participants (81.4%) were single, and 50.7% had a secondary school level of education. Half of the included participants had 5–7 family members, while one-third had more than 7 family members. About 35% of the participants were from the Western region, 44.9% were from the Central region, and the remaining participants were from the Eastern and Southern regions. The majority of participants were students (62.2%), and 198 participants (29%) were employees in the public and private sectors. Two-thirds of the included participants (*n* = 454) had monthly incomes ranging from 500 to 2,000 LD.

### PROMIS Depression, Perceived Stress, and Sleep Disturbance

The overall median PROMIS Depression scale total score was 20.00 (out of 40.00), with a significantly higher median total score for females compared with males (22 vs. 18, *p* = 0.001). A higher total score was observed among the 18–29 age group (median = 21), followed by the 30–39 age group (median = 17). In terms of stress, more than half of the respondents (52.2%, *N* = 360) reported moderate stress, while high stress was reported by 17% of the study sample (*N* = 117). A previously published article from China documented lower stress percentages in 7,143 respondents during the COVID-19 outbreak (Cao et al., 2020) which stated that 0.9, 2.7, and 21.3% had severe, moderate, or mild stress, respectively. This variation can be explained by the different populations studied, as they included only Chinese college students without differentiating between students at higher education levels from younger students, who experienced less stress. Another study conducted by Shangguan et al. (2020) with 1,134 participants showed that 8.0% of the included participants had moderate to severe anxiety, while 7.4% reported somatization.

The overall median perceived stress scale total score was 18.00, with a significantly higher median total score for females compared with males (20 vs. 15, *p* = 0.001). Notably, stress symptoms were more prevalent among the younger age groups. A higher total score was observed among the 18–29 age group (median = 19) followed by the 30–39 and 40–49 age groups (median = 17 for each). Our findings provided data that improves our understanding of the prevalence and influencing factors that have contributed to the psychosocial burdens of the Libyan public during the COVID-19 outbreak. This survey highlights that the levels of depression and perceived stress by the general public are alarming and need immediate attention. This high prevalence of stress among the Libyan public can be explained by uncertainty over the COVID-19 pandemic's progression as well as the public's fear of getting infected and of transmitting the virus to their loved ones. This fear is exacerbated by the lack of consistent guidance from the authorities as well as the impact of self-isolation on the psychological well-being of respondents.

Moreover, as reported by Wells et al. (2011), the high levels of depression and psychological distress may also be associated with the civil war, which has taken place in Libya for about 10 years. Another possible explanation for the percentage of high stress among the public could be due to the insufficient trust of the public in both local and central governments to manage the pandemic and prevent further waves of the virus (87.26% were not confident in their local government in managing the pandemic, and 89.31% were not confident in the central government to prevent a further outbreak of the coronavirus). This poor confidence in the authorities fuels fear and panic among the public, which has an adverse impact on psychosocial well-being. This finding is supported by the literature, where fear of being infected and that the pandemic was hard to control were sources of mental burdens (Huang and Zhao, 2020; Raza et al., 2020).

The overall median PROMIS Sleep Disturbance scale total score was 56.30, with a significantly higher median total score for females than males (56.3 vs. 54.3, *p* = 0.028). The highest total score was observed among the 18–29 age group (median= 57.30), followed by the 30–39 and 50–59 age groups (median = 54.30 and median = 52.20, respectively). This finding reflects the high responsibilities that Libyan females have in taking care of their families and managing the risk of the novel COVID-19 virus, which adversely impacts their sleep. There are, however, other possible explanations for these findings. Libyan females are generally silent in terms of expressing their psychological distress and seeking medical advice due to the social and cultural norms of the Libyan community. This consequently may have a serious impact on their mental health, and of course their sleep quality.

Prior studies have noted similar findings in regard to stress, depression, and sleep disturbances. For example, a study from China by Fu et al. (2020) reported the incidence rates of anxiety symptoms (27.5%), depressive symptoms (29.3%), and sleep disorders (30.0%). Moreover, in Ren et al.'s study (Ren et al., 2020), depressive and anxiety symptoms were 18.8 and 13.3%, respectively for the included 1,172 participants. Huang et al. (2020) assessed the prevalence of anxiety, sleep disturbances, and somatization among 1,172 participants from 125 Chinese cities, and found that 33.02% had anxiety symptoms, 7.59% had somatization, and 24.66% had sleep disturbances (insomnia). Similarly, Zhou et al. (2020) conducted a study on 1,099 participants from the general population and found that 47.6, 33.8, and 25.1%, had depressive symptoms, anxiety, and insomnia, respectively. Another Chinese cross-sectional study of 7,236 participants documented anxiety disorder in 35.1% of the included participants, depressive symptoms in 20.1%, and sleep disturbances in 18.2% (Huang and Zhao, 2020). The authors noted significantly higher rates of anxiety disorder as well as depressive symptoms among younger populations. The variations among the published articles and our study's findings may be due to the different populations studied as well as the different times of assessment, as most of the published studies were conducted during the early phase of the COVID-19 pandemic.

Moccia et al. (2020) conducted a cross-sectional study on 500 participants in Italy, and reported mild psychological distress among 19.4% of the included participants, while 18.6% had moderate to severe distress. Another study conducted by Mazza et al. (2020) documented self-rated proportions of 31, 42, and 40% for depression, anxiety, and insomnia, respectively. They also found that females reported significantly higher rates of anxiety and depression than males, despite the decreased baseline inflammatory markers. An study from Australia on 5,071 participants showed that 62, 50, and 64% had depressive symptoms, anxiety symptoms, and increased stress levels, respectively (Newby et al., 2020), while in Spain, a study on 3,480 participants from the general population showed that 18.7 and 21.6% had depression and anxiety symptoms, respectively (González-Sanguino et al., 2020). Meanwhile, a national assessment of at-risk French populations that included 1,771 participants found that 38.06% of participants had psychological distress (Chaix et al., 2020).

In the current study, we have reported higher levels of stress, depression, and sleep disturbances than what has been documented in the literature. A possible explanation for these results may be due to the protracted armed conflicts, the fragile political and economic state of Libya, and the uncertainty over the progression of the COVID-19 pandemic, which altogether have had a serious impact on Libyans' psychological and social well-being (The United Nations Office for the Coordination of Humanitarian affairs, 2020). Another possible explanation for these findings can be attributed to the fact that there is a lack of adequate mental health screening programs that target vulnerable populations. In fact, seeking mental health consultations is highly stigmatized in Libya and needs further attention.

### Self-Efficacy Total Scores

The construct of self-efficacy is grounded in the Social Cognitive Theory, which refers to personal beliefs in one's capabilities. Generally, self-efficacy determines how individuals feel, think, motivate themselves, and behave in certain ways. Research has shown that high self-efficacy is instrumental in self-care behaviors in many conditions because it enhances emotional well-being and improves coping capabilities (Bidzan et al., 2020). In the current study, the overall median self-efficacy total score was 6.67 (out of 10), with a significantly higher median total score for males than females (7 vs. 6.33, *p* = 0.001). Higher total scores were observed among the 40–49 and 50–59 age groups (median = 7.00 for each), followed by the 30–39 age group (median = 6.92).

Additionally, a significant negative correlation was reported between self-efficacy and gender (*P* < 0.001), where female respondents reported less self-efficacy than their male counterparts. A growing body of literature suggests a correlation between self-efficacy and psychological well-being (Cohen et al., 1983; Regmi et al., 2016; Wolf et al., 2020), as was the case in our study. However, the direction of causal relationships cannot be established using cross-sectional designs, and therefore further research is needed to better address this issue. A previous study from China on 180 medical staff showed that self-efficacy and sleep quality were correlated with social support for healthcare providers, while anxiety and depression were negatively associated. Anxiety levels were directly linked to stress levels, which had a negative effect on self-efficacy and the quality of sleep. Anxiety, stress, and self-efficacy were found to be mediators of social support and quality of sleep (Xiao et al., 2020). A recent systematic review of 43 studies documented that compared to prior to the COVID-19 outbreak, studies of the general population documented reduced psychological well-being as well as elevated anxiety and depression scores. Certain factors were associated with such findings in that review, which included female sex and reduced self-related health (Vindegaard and Benros, 2020).

After multivariate regression analyses, we found that female participants may be potentially at higher risks for stress and depression than their male counterparts. These findings may be related to the body's normal protective response to the stress and depression caused by the pandemics as reported in the literature (Maunder et al., 2003). Furthermore, participants aged between 40 and 49 years were more likely to have poorer sleep quality than the other segments of the population. Our findings, however, are consistent with previous studies in China (Huang and Zhao, 2020) and Canada (Guadagni et al., 2020). In addition, we assessed the total self-efficacy scores among participants and found that female participants were more likely to have lower self-efficacy than males. Such findings may underscore the importance of interventions that target mental health, especially for females during the pandemic. Improving the public's self-efficacy during the pandemic may be helpful to their mental health.

Given the delay in providing vaccinations to the public in Libya and the difficulty among public to cope with the challenges posed by the pandemic, it is expected that the pandemic-related psychosocial crisis will continue for several months at best. In order to better address these mental issues, it is imperative for government and non-government organizations to take a series of steps to develop programs that target the public to help them cope with the psychosocial challenges they face. These programs can be done through releasing consistent and evidence-based guidelines for mental health intervention during COVID-19, providing telephone and online psychological counseling, and creating reliable resources to educate the public about the possible mental problems.

### Housing and Living Situations of the Respondents

Based on our findings, a little more than half of the study's respondents (56.4%, *N* = 385) had between 6 and 10 people living in the same home, while 28.9% of the study sample shared the same household with more than 10 people (*N* = 198). We also found that 42.6% of the respondents (*N* = 291) had moved or changed their residence because of the war. However, only 2.1% (*N* = 14) had changed their residence because of the pandemic. Research shows that the quality of one's living situation has a significant influence on psychological well-being, and can be a risk factor for further development of psychological burdens. In Wathelet et al. (2020), a total of 69,054 French students were studied, and the authors documented that having at least one mental health condition was linked to low-quality housing [odds ratio = 2.30; 95% confidence interval (2.06–2.57); *P* < 0.001]. Another study on 389 frontline physicians in Pakistan showed that the presence of children among members of a household was one of the independent risk factors for anxiety/depression among healthcare workers (adjusted odds ratio = 1.58; 95% confidence interval 1.00–2.50) (Amin et al., 2020). Another study on students from an under-resourced public university showed that lower household savings enhanced anxiety symptoms (odds ratio = 1.3; 95% confidence interval 1.0–1.6), but did not increase the risk of depressive symptoms (Rudenstine et al., 2021).

It has been acknowledged that a good housing environment is central to social and psychological well-being because it is closely related to the way persons live, interact, organize their daily routines, and have a sense of safety and security. In this regard, the housing and living situation in Libya is problematic for several reasons. As of December 2019, over 300,000 people had been displaced because of the war (Human Rights Watch, 2020), with most of them living with relatives and residing in a variety of housing arrangements, such as rented apartments, schools, and mosques. This poor housing situation has been imposed by the intermittent military conflicts in many parts of the country for nearly 10 years. By mid-2020, the World Health Organization urged all armed groups in Libya to accept the ceasefire in order to help combat the dramatic increase in the country's COVID-19 cases. At the time of writing this report (January 2021), the armed conflicts are still happening, which might force more and more civilians to flee their homes, which may in turn cause further deterioration regarding psychosocial burdens among the public in Libya.

### Strengths and Limitations of the Study

We circulated this study throughout 21 municipalities across the country, and data were collected from a representative sample of the Libyan public. Although female respondents were a little higher in representation (58.4%) than the actual demographic figures for Libya, our results are similar to previous surveys in other countries such as Germany and the United Kingdom, where female respondents were higher in representation than males at 70.7 and 84.5%, respectively (Bauerle et al., 2020). One possible explanation for this observation is that female respondents may have different values regarding participating in online research activities than males. As reported in previous research, male participants are more likely to answer survey questions if they receive a reminder or a reward (Saleh and Bista, 2017); this study, however, was voluntary, and we did not provide a reward for participation.

There is also a limitation inherent to the cross-sectional nature of the study, such as the inability to assess causation and temporal relationship between the independent and dependent variables. Therefore, we recommend future research to consider longitudinal research designs to better address these issues. Also, the sample may not represent the entire Libyan public as it involved snowball sampling technique, and mostly it was only available for those who have social media and can fill online surveys. This was evident by having a sample of mostly relatively young single participants. However, based on the demographic figures in Libya, nearly 60% of the population is under 30 (Larémont, 2012). Thus, we believe that the included sample represent the majority of Libyan people.

The other limitation of our study was that we did not control for physicians, as physicians in particular and healthcare workers in general report higher scores for depressive and psychological burdens compared to the general public. However, given the busy schedule of physicians during the pandemic, it is unlikely that the number of physician respondents would have influenced the findings of this study. Despite these limitations, however, this study is the first country-wide survey to provide valuable data about the psychosocial impact of the COVID-19 pandemic in Libya.

## Conclusions and Recommendations

Evidently, the world is confronting perhaps the most difficult challenge of our lifetimes because of the recurrent waves of the novel COVID-19 virus, which has now spread to virtually every country in the world. The impacts of the COVID-19 pandemic are becoming more prominent in various measurements like social, mental, and economic impacts. COVID-19 flare-ups have changed the current living situation of millions of people due to mandatory social isolation, fear of contracting the virus through human-to-human contact, and closures of educational, business, and recreational facilities. As reported by several researchers, the present circumstances could have extreme effects on individuals' psychosocial health and well-being (Rajkumar, 2020). This finding was proved true based on our survey, as the scores for all psychosocial variables were significantly high, especially for perceived stress and sleep disturbances, which are two critical indicators of the overall mental health of the public. During this era of COVID-19, there is a paucity of evidence about the prevalence rate as well as the insomnia-associated risk factors within the general population. Previously published articles (Cellini et al., 2020; Zhang et al., 2020; Lin et al., 2021) have mainly assessed them among either healthcare workers or specific populations, and thus there is a need to assess psychological symptoms as well as insomnia prevalence rates and associated risk factors globally.

Furthermore, the situation in Libya has been escalated by the political instability and military conflicts that have resulted in the deterioration of public services, including those for basic needs. All these stressors and enforcements have put extra pressure on civilians' shoulders and have imposed further burdens on them, their families, and the community at large. All the above-mentioned restrictions have the potential to increase the psychosocial burdens of the public in Libya, as evidenced by this study.

To conclude, the outcomes of this research suggest increased prevalence rates of stress, depression, and sleep disturbances as well as COVID-19-related fear during the pandemic, especially among young females (18–29 years old). The findings also suggest that self-efficacy was also lower among young female respondents. Most participants reported an increased smoking frequency, and nearly one-third displayed less healthy eating habits during the pandemic. This study identified significant social and psychological burdens of the Libyan public during the COVID-19 pandemic; thus, it is crucial to provide safe, low-barrier interventions to help burdened individuals. This study makes a significant contribution in providing essential data on the psychological and social impacts on the Libyan population caused by the COVID-19 pandemic. Such information is critical for gathering much-needed evidence on emerging psychosocial health problems and determining the need for targeted approaches in order to support the Libyan people.

## Data Availability Statement

The raw data supporting the conclusions of this article will be made available by the authors, without undue reservation.

## Ethics Statement

The studies involving human participants were reviewed and approved by The Research Ethics Boards of the following institutions: The University of Misrata, the Misrata Medical Center, the Misrata Cancer Center, & the University of Tripoli. The patients/participants provided their written informed consent to participate in this study.

## Author Contributions

AJ drafted the first version of the manuscript and approved the final version. All authors contributed equally to the planning, data collection, and manuscript preparation, read and approved the manuscript before submission.

## Conflict of Interest

The authors declare that the research was conducted in the absence of any commercial or financial relationships that could be construed as a potential conflict of interest.

## Publisher's Note

All claims expressed in this article are solely those of the authors and do not necessarily represent those of their affiliated organizations, or those of the publisher, the editors and the reviewers. Any product that may be evaluated in this article, or claim that may be made by its manufacturer, is not guaranteed or endorsed by the publisher.
