# Supplementary material for: Psychosocial Impact of COVID-19 Pandemic in Libya: A Cross-Sectional Study
Source: Front Psychol. 2021 Aug 17;12:714749. doi: 10.3389/fpsyg.2021.714749 (PMC8416112; doi:10.3389/fpsyg.2021.714749)
Supplement: Supplementary file 1 [file Table_1.pdf]

Model Coefficients – The Perceived Stress				
**Predictor**	**Estimate**	**SE**	**t**	**p**
**Intercept**	16.83	1.63	10.32	< .001
**Age:**				
18-29		**REFERENCE**		
30-39	-0.68	1.25	-0.54	0.59
40-49	0.03	1.54	0.02	0.98
50-59	-2.24	1.93	-1.16	0.25
60+	-5.35	4.27	-1.25	0.21
**Gender:**				
Male		**REFERENCE**		
Female	3.46	0.64	5.42	< .001
**Marital status:**				
Single		**REFERENCE**		
Married	-1.99	1.07	-1.86	0.06
Divorced	-1.16	3.57	-0.32	0.75
Widow	-1.23	5.58	-0.22	0.83
**Education:**				
Primary		**REFERENCE**		
Secondary	0.44	1.59	0.28	0.78
Bachelor	-0.26	1.64	-0.16	0.87
Master	-0.46	2.16	-0.21	0.83
Doctor or more	-1.59	2.30	-0.69	0.49
Vocational training	0.57	3.27	0.17	0.86
No formal education	-2.16	3.45	-0.63	0.53
**Occupation:**				
Student		**REFERENCE**		
Public sector employee	1.07	1.18	0.91	0.36
Private sector employee	-1.17	1.02	-1.15	0.25
Not working, unemployed, housewife	1.94	1.30	1.50	0.14
Retired	3.32	3.97	0.84	0.40

Model Coefficients – Sleep Quality				
**Predictor**	**Estimate**	**SE**	**t**	**p**
**Intercept**	57.71	2.30	25.05	< .001
**Age:**				
18-29		**REFERENCE**		
30-39	-0.03	1.77	-0.02	0.99
40-49	-4.58	2.18	-2.10	0.04
50-59	-2.69	2.72	-0.99	0.32

60+	-5.96	6.03	-0.99	0.32
**Gender:**				
Male		**REFERENCE**		
Female	0.80	0.90	0.89	0.37
**Marital status:**				
Single		**REFERENCE**		
Married	-2.77	1.51	-1.84	0.07
Divorced	-1.69	5.04	-0.34	0.74
Widow	7.00	7.88	0.89	0.38
**Education:**				
Primary		**REFERENCE**		
Secondary	-1.61	2.25	-0.72	0.47
Bachelor	-2.58	2.31	-1.12	0.27
Master	-1.97	3.05	-0.64	0.52
Doctor or more	-5.76	3.25	-1.77	0.08
Vocational training	4.09	4.62	0.88	0.38
No formal education	-6.65	4.87	-1.37	0.17
**Occupation:**				
Student		**REFERENCE**		
Public sector employee	-0.29	1.66	-0.17	0.86
Private sector employee	-0.24	1.44	-0.16	0.87
Not working, unemployed, housewife	2.55	1.83	1.39	0.16
Retired	-0.59	5.61	-0.11	0.92

Model Coefficients - Depression

**Predictor**	**Estimate**	**SE**	**t**	**p**
**Intercept**	21.83	1.76	12.42	< .001
**Age:**				
18-29		**REFERENCE**		
30-39	0.14	1.35	0.10	0.92
40-49	-0.52	1.66	-0.31	0.75
50-59	-2.11	2.07	-1.02	0.31
60+	-2.59	4.60	-0.56	0.57
**Gender:**				
Male		**REFERENCE**		
Female	2.04	0.69	2.97	0.00
**Marital status:**				
Single		**REFERENCE**		
Married	-4.47	1.15	-3.89	< .001
Divorced	1.87	3.85	0.49	0.63

Widow	1.71	6.02	0.28	0.78
**Education:**				
Primary		**REFERENCE**		
Secondary	-1.37	1.71	-0.80	0.43
Bachelor	-2.55	1.76	-1.45	0.15
Master	-4.06	2.33	-1.74	0.08
Doctor or more	-4.16	2.48	-1.68	0.09
Vocational training	-4.76	3.53	-1.35	0.18
No formal education	-2.52	3.72	-0.68	0.50
**Occupation:**				
Student		**REFERENCE**		
Public sector employee	1.42	1.27	1.12	0.26
Private sector employee	0.47	1.10	0.43	0.67
Not working, unemployed, housewife	2.51	1.40	1.80	0.07
Retired	3.04	4.28	0.71	0.48

Model Coefficients – Self-efficacy

**Predictor**	**Estimate**	**SE**	**t**	**p**
**Intercept**	6.86	0.40	17.32	< .001
**Gender:**				
Male		**REFERENCE**		
Female	-0.53	0.16	-3.45	< .001
**Monthly income:**				
Less than 500		**REFERENCE**		
500 to 1000	-0.31	0.40	-0.76	0.45
1000 to 2000	-0.14	0.40	-0.34	0.73
2000 to 5000	-0.06	0.42	-0.14	0.89
more than 5000	0.61	0.46	1.31	0.19
